# Impact of timing and severity of COVID-19 infection in pregnancy on intrauterine fetal growth- a registry-based study from Qatar

**DOI:** 10.1371/journal.pone.0288004

**Published:** 2023-06-30

**Authors:** Thomas Farrell, Fathima Minisha, Salwa Abu Yaqoub, Abubaker Abdel Rahim, Mai Omar, Huda Ahmed, Stephen Lindow, Merlin Rajam Abraham, Mahmoud Gassim, Nader Al-Dewik, Shamsa Ahmed, Hilal Al-Rifai, Q-precious group

**Affiliations:** 1 Department of Obstetrics and Gynecology, Women’s Wellness and Research Centre, Hamad Medical Corporation, Doha, Qatar; 2 Director of Master’s Projects, Coombe Women and Infants University Hospital, Dublin, Ireland; 3 Department of Research, Women’s Wellness and Research Centre, Hamad Medical Corporation, Doha, Qatar; 4 Department of Pharmacology, Women’s Wellness and Research Centre, Hamad Medical Corporation, Doha, Qatar; 5 Chief Executive Officer, Women’s Wellness and Research Centre, Hamad Medical Corporation, Doha, Qatar; University of Cambridge, UNITED KINGDOM

## Abstract

**Background:**

The novel coronavirus disease (COVID-19) pandemic has impacted pregnant women, increasing maternal and neonatal morbidity. The placenta is a potential target for the pathophysiological processes due to the increased thrombotic inflammatory activation and inadequate uteroplacental perfusion and oxygenation, potentially causing intrauterine growth restriction. This study investigates the impact of gestational age at diagnosis of COVID-19 and the presence of symptoms on intrauterine fetal growth in pregnant women.

**Methods:**

A retrospective review of COVID-19 positive pregnant women in Qatar from March 2020 to March 2021 was conducted. They were divided based on trimester of pregnancy in which they were infected. The outcomes included birthweight, customised fetal birthweight centiles, small for gestational age (SGA) baby and daily growth increments, compared between the trimesters and between symptomatic and asymptomatic women.

**Results:**

In our cohort, 218 women (20.5%) were infected in the first trimester, 399 (37.5%) in the second and 446 (42%) in the third. Women in the second trimester were significantly younger and symptomatic. Women infected in the first trimester were least likely to have diabetes. The mean birthweight, risk of SGA (11.5% vs 10% vs 14.6%, p = 0.302), and median customized growth centiles (47.6% vs 45.9% vs 46.1%)were similar between the groups. Symptomatic women had significantly lower mean birthweight (3147 gms vs 3222 gms) and median birthweight centiles (43.9% vs 54.0%)compared to the asymptomatic (p<0.05 for both). In women infected within 20 weeks of gestation, a delay in daily fetal growth increments was noted with symptomatic disease, although not statistically significant.

**Conclusion:**

This study shows that women with symptomatic disease had lower birth centiles and birth weights. This was regardless of the gestational age at which they were infected. Early symptomatic disease seems to have an impact on fetal growth velocity; however, larger studies are needed to corroborate these findings.

## Introduction

### Background and rationale

Coronavirus Disease-19 (COVID-19), which is caused by the severe acute respiratory syndrome coronavirus 2 (SARS-CoV-2) virus, was declared a global pandemic at the start of 2020 by the World Health Organization (WHO). Since then, a plethora of articles has attempted to determine the relationship between the virus and pregnancy and neonatal outcomes. As a result, pregnant women were designated as a vulnerable group based on initial reports of increased risk of stillbirth, preterm birth and fetal growth restriction (FGR). Evidence suggests that infection in the later stages of pregnancy is associated with more severe adverse obstetric and neonatal outcomes; however, this may be due to a lack of data concerning COVID-19 infection in the early stages of pregnancy [1]. In addition, those pregnant women with COVID-19 who were symptomatic carried a greater risk of adverse outcomes than asymptomatic women [2].

There is increasing evidence that COVID-19 infection is vertically transmitted [3, 4]. The placenta seems to become a potential target for pathophysiological processes that could affect pregnancy outcomes in COVID-19 patients due to increased thrombotic risk and inflammatory activation [5–7]. As placental oxygenation is central in regulating fetoplacental angiogenesis, inadequate uteroplacental perfusion and oxygenation due to placental ischemia and abnormal angiogenesis are considered the primary cause of fetal intrauterine growth restriction and decreased fetal growth velocity [8, 9]. Although the pathophysiological mechanisms that cause fetal growth restriction likely start in the first half of pregnancy, the clinical signs of ischemic placental disease might become apparent late in pregnancy.

### Study aims

The primary aim of this study was to assess the impact of gestational age at diagnosis of COVID-19 and symptoms status on fetal growth (assessed using birthweight and customised birthweight centiles) in a cohort of pregnant women delivering in a large tertiary maternity unit in Qatar. The secondary aim was to explore the differences in fetal growth and velocity (assessed using birthweight, customised birthweight centiles and customised fetal growth velocity z scores) according to symptom status in a subgroup of women who were infected in the first 20 weeks of pregnancy.

## Materials and methods

### Study design and setting

The Q-PRECIOUS is an active national perinatal registry consisting of women diagnosed with COVID-19 infection during their pregnancies from the beginning of the pandemic. The registry includes all women receiving maternity care within the State of Qatar (public or private sector). The registry was approved by the Medical Research Centre (MRC), Hamad Medical Corporation (HMC), Qatar (MRC- 01-21-122) and by the Hamad Medical Corporation (HMC) Institutional Review Board (IRB), with a waiver of consent provided. Since all the data for this study was extracted from the registry, additional approval was not required as per the corporation MRC policies.

The registry consists of data extracted from retrospective chart reviews of women infected with COVID-19 during their pregnancies, via the Cerner Millennium^®^ patient electronic health records. The list for the registry was sourced from the Ministry of Public Health COVID-19 records. We accessed the anonymized data of women infected between March 2020 and March 2021 after prior approval of our study from the registry lead. We did not extract or use any patient identifiable variables like hospital number, name, or date of birth for the analysis. The data cleaning and analysis was performed in the Department of Research, Women’s Wellness and Research Centre, the largest tertiary maternity centre in the country and location of the registry database.

### Study participants

The diagnosis of COVID-19 infection was made following a positive RT-PCR (reverse transcription polymerase chain reaction) naso-oropharyngeal swab test analysed in the central Department of Laboratory and Pathology, HMC. Women were screened either due to clinical suspicion of COVID-19, as part of contact tracing or during screening for elective hospital admission. Pregnancy needed to be confirmed either clinically and/or by a urine or blood pregnancy test and/or by a pelvic ultrasound at the time of infection.

Women having a miscarriage (delivery below the period of viability ≤24 completed weeks of gestation as per local hospital guidelines) were excluded as the main aim was to assess the effect of Covid-19 on intrauterine fetal growth. Additionally, a subgroup of women infected in the first 20 weeks of gestation were included in the secondary analysis. This group included all women who had undergone a minimum of two third trimester ultrasounds for fetal biometry, a minimum of two and a maximum of six weeks apart (in order to ensure both scans are in the third trimester). This allowed us to investigate women who got infected early but carried on their pregnancies beyond 34 weeks.

### Variable definitions and data source

As part of the national COVID-19 prevention and control policies, any person testing positive was contacted by a health professional enquiring about symptoms. Pregnant women reporting any WHO-advised disease-related symptoms [10] were grouped in the symptomatic group, and the remaining women formed the asymptomatic comparison group. The symptoms reported ranged from common ones such as cough, fever, myalgia and sore throat to more severe rarer symptoms such as shortness of breath and chest pain. The gestational age at diagnosis of COVID-19 infection was determined from the estimated delivery date, based on either a dating ultrasound performed in the first trimester of pregnancy or, when this is not available, the last menstrual period (LMP). Gestational age in completed weeks at the time of COVID-19 infection was then categorised into- the first trimester (up to 13 completed weeks), second trimester (14–27 weeks), and third trimester (28–40 weeks), forming the main exposure groups. The maternal age, body mass index (BMI), parity and country of nationality were extracted from medical records.

Preexisting maternal medical conditions such as asthma, chronic hypertension, and diabetes were recorded as binary variables. Medical conditions with counts less than five were grouped into the ’other medical illness’ category to safeguard patient confidentiality. They included diseases affecting the cardiovascular system, haematological system, autoimmune conditions, renal diseases, gastrointestinal disorders, malignancy, etc. Pregnancy variables such as hypothyroidism, gestational diabetes, gestational hypertension (including preeclampsia and eclampsia), anaemia and fetal gender were also collected.

The main outcome variables included birthweight- defined as the weight measured in grams within the first hour of birth, and a customised birthweight centile which adjusted the birthweight for maternal parity, ethnicity, height and weight, fetal sex, fetal weight and gestational at birth, calculated using the software program GROW (Gestation Network; Birmingham, UK) [11–14]. The babies were further classified as small for gestational age (SGA if centiles ≤10%) and large for gestational age (LGA if centiles ≥90%).

The subgroup of women infected within the first 20 weeks of gestation were selected because during this period, the primary and secondary waves of placental invasion occur and infection during this period has a higher probability of adverse effects on the placental circulation [15]. An additional outcome parameter for this subgroup of women, was fetal growth velocity z score, calculated using third trimester ultrasound scans. The estimated fetal weight (EFW) reported in each ultrasound scan and the interval, in days, between scan 1 and scan 2 were used to calculate the fetal growth per day over the time period for each baby. The EFW was calculated using the Hadlock’s formula including ultrasound estimation of fetal head circumference, biparietal diameter, femur length and abdominal circumference [16]. Individual growth increment Z scores (standardised scores) were calculated using the observed growth for each baby and the mean and standard deviation (SD) for the entire subgroup [17].

### Statistical analysis

The continuous demographic variables were represented as mean ± SD or median ± range according to the assessment of normality (done by visual assessment- histograms; and Shapiro Wilk test SW). The categorical variables were represented as frequency and percentage. The baseline variables were compared among the three trimester groups using ANOVA/ Kruskal Wallis test for continuous and Chi-Square/Fishers exact test for categorical as appropriate.

The birthweight and gestational age at delivery in days were compared between the three exposure groups using one-way ANOVA. The customised birthweight centiles were compared using Kruskal-Wallis test. The categorical outcomes (proportion of preterm birth, SGA and LGA) were compared using Chi-square test. Further adjusted analysis was done only if statistically significant results were noted in the univariate analysis.

The women were then divided according to symptom status (symptomatic vs asymptomatic disease), separately for the entire cohort and the subgroup getting infected within 20 weeks. The outcomes were compared using Student t-test/ Mann Whitney U test for continuous variables or Chi-square/Fishers for categorical variables. The significance level was set at p-value less than 0.05. All analysis was conducted using Statistical Package for Social Sciences, version 28 (IBM Corp., New York).

## Results

There were 1063 women infected with COVID-19 in pregnancy; 218 (20.5%) were infected in the first trimester, 399 (37.5%) in the second and the remaining 446 (42%) in the third trimester. Table 1 outlines the characteristics of the women according to the trimester in which they were infected.

**Table 1:** Comparison of baseline and pregnancy factors according to trimester of infection.

Baseline and Pregnancy risk factors		1^st^ trimester infection N = 218	2^nd^ trimester infection N = 399	3^rd^ trimester infection N = 446	p-value
**Maternal age- years** (Mean, SD)*		30.6 (±5.5)	30.4 (±5.3)	31.4 (±5.3)	**0.018**
**Maternal BMI- kg/m**^**2**^ (Mean, SD)*		29.2 (±5.6)	30.4 (±6.3)	31.1 (±13.0)	0.057
**Maternal parity** (Mean, SD)*		2.1 (±1.7)	1.9 (±1.6)	2.0 (±1.7)	0.347
**Qatari Nationality** (Number, %N)		171 (78.4%)	284 (71.2%)	313 (70.2%)	0.069
**COVID-19 symptoms** (Number, %N)	**Asymptomatic**	68 (31.2%)	107 (26.8%)	164 (36.8%)	**0.001**
	**1 symptom**	38 (17.4%)	70 (17.5%)	97 (21.7%)	
	**≥ 2 symptoms**	112 (51.4%)	222 (55.6%)	185 (41.5%)	
**Fever** (Number, %N)		69 (31.7%)	138 (34.6%)	135 (30.3%)	0.400
**No co-morbidity** (Number, %N)		111 (50.9%)	177 (44.4%)	195 (43.7%)	0.187
**Hypertensive disease** (Number, %N)		11 (5.1%)	11 (2.8%)	12 (2.7%)	0.220
**Hypothyroidism** (Number, %N)		21 (9.6%)	38 (9.5%)	41(9.2%)	0.979
**Diabetes** (Number, %N)	**None**	150 (68.8%)	253 (63.9%)	284 (63.7%)	**0.040**
	**Gestational diabetes**	56 (25.7%)	135 (33.8%)	151 (33.9%)	
	**Preexisting diabetes**	12 (5.5%)	9 (2.3%)	11 (2.5%)	

SD- standard deviation, BMI- body mass index, N- number of women in each exposure group, kg/m^2^- kilograms/metres^2^, ^ analysed using ANOVA- Analysis of variance; p<0.05 considered evidence against null hypothesis of no difference.

There was a significant difference in symptomatology between the groups, with women infected with COVID-19 in the second trimester more likely to have two or more symptoms and least likely to be asymptomatic. Additionally, women in the second trimester were significantly younger (30.4 years old) compared to 30.6 and 31.4 years old in the first and third trimesters, respectively. Women infected in the first trimester were more likely to have preexisting diabetes but less likely to develop gestational diabetes during pregnancy. There was no statistically significant difference between the groups’ maternal BMI, parity, nationality, hypertension, hypothyroidism, or cholestasis of pregnancy.

The comparison of outcomes between the three groups is shown in Table 2. The gestational age at birth in days and birthweight in grams were normally distributed (SW test p>0.05). There was no difference noted in gestational age or birthweight between the groups. The customised birthweight centile was compared non-parametrically- no difference was noted between the trimesters. More than 12% of the cohort delivered SGA babies. Those infected in the third trimester had a higher chance of having an SGA baby, but this did not reach statistical significance. The risk of LGA babies (overall 11.9%) and preterm birth (overall 10.6%) did not vary according to the trimester of acquiring COVID-19.

**Table 2:** Comparison of outcome variables among the exposure groups.

Outcome variables		Total N = 1063	1^st^ trimester infection N = 218	2^nd^ trimester infection N = 399	3^rd^ trimester infection N = 446	P value
**Gestational age at delivery (days)** Mean (SD)*		267.7(± 13.2)	267.7 (± 12.6)	267.6 (± 13.3)	267.9 (± 13.8)	0.947
**Birthweight in grams**; Mean (SD)*		3171 (± 536)	3170 (± 541)	3173 (± 531)	3169 (± 537)	0.994
**Customised birthweight centile**; Median (Range)**		47.0 (0–100)	47.6 (0–100)	45.9 (0–100)	46.1 (0–100)	0.752
**Birthweight centile**; Number, %N	**<10** ^**th**^ **- SGA**	130 (12.2%)	25 (11.5%)	40 (10.0%)	65 (14.6%)	0.302
	**10-90** ^**th**^	807 (75.9%)	164 (75.2%)	310 (77.7%)	333 (74.7%)	
	**>90** ^**th**^ **- LGA**	126 (11.9%)	29 (13.3%)	49 (12.3%)	48 (10.8%)	
**Preterm births**; Number, %N		113 (10.6%)	25 (11.5%)	36 (9.0%)	52 (11.7%)	0.418

SD- standard deviation, IQR- interquartile range, N- number of women in each exposure group, SGA- small for gestational age, LGA- large for gestational age,

* analysis done using ANOVA- Analysis of variance;

**- analysis done using Kruskal Wallis test;

p<0.05 considered evidence against null hypothesis of no difference.

There were 724 women in the entire cohort (68%) who developed symptomatic COVID-19. The outcomes were also compared between asymptomatic and symptomatic women (Table 3). Mean maternal age and maternal BMI were significantly higher in the symptomatic group. Furthermore, the mean birthweight (3147 ± 537 vs 3222 ± 528; p<0.05) and median customised birth centiles (43.9% vs 54.0%; p = 0.004) were significantly lower in this group than in asymptomatic women.

**Table 3:** Comparison between symptomatic and asymptomatic COVID.

Variables in 1063 women		Asymptomatic (N = 339)	Symptomatic (N = 724)	p value
**Maternal age, years**, Mean (±SD)*		30.4 (± 5.2)	31.1 (± 5.4)	**0.047**
**Maternal BMI, kg/m**^**2**^**,** Mean (±SD) *		29.5 (± 6.2)	30.9 (± 10.9)	**0.028**
**Birthweight in grams**, Mean (±SD)*		3222 (± 528)	3147 (± 537)	**0.033**
**Customised birthweight centile**; Median (Range)**		54.0 (0.2–100)	43.9 (0–100)	**0.004**
**Birthweight centile** Number, %N	**<10th centile (SGA)**	36 (10.6%)	94 (13.0%)	0.190
	**10-90th centile**	255 (75.2%)	552 (76.2%)	
	**>90th centile (LGA)**	48 (14.2%)	78 (10.8%)	
**Preterm birth**; Number, %N		35 (10.3%)	78 (10.8%)	0.825

*- analysis done using Student t-test;

**- analysis done using Mann Whitney U test;

BMI- body mass index; SD- standard deviation; IQR- interquartile range; SGA- small for gestational age; LGA- Large for gestational age; p<0.05 considered evidence against null hypothesis of no difference

**Fig 1:**
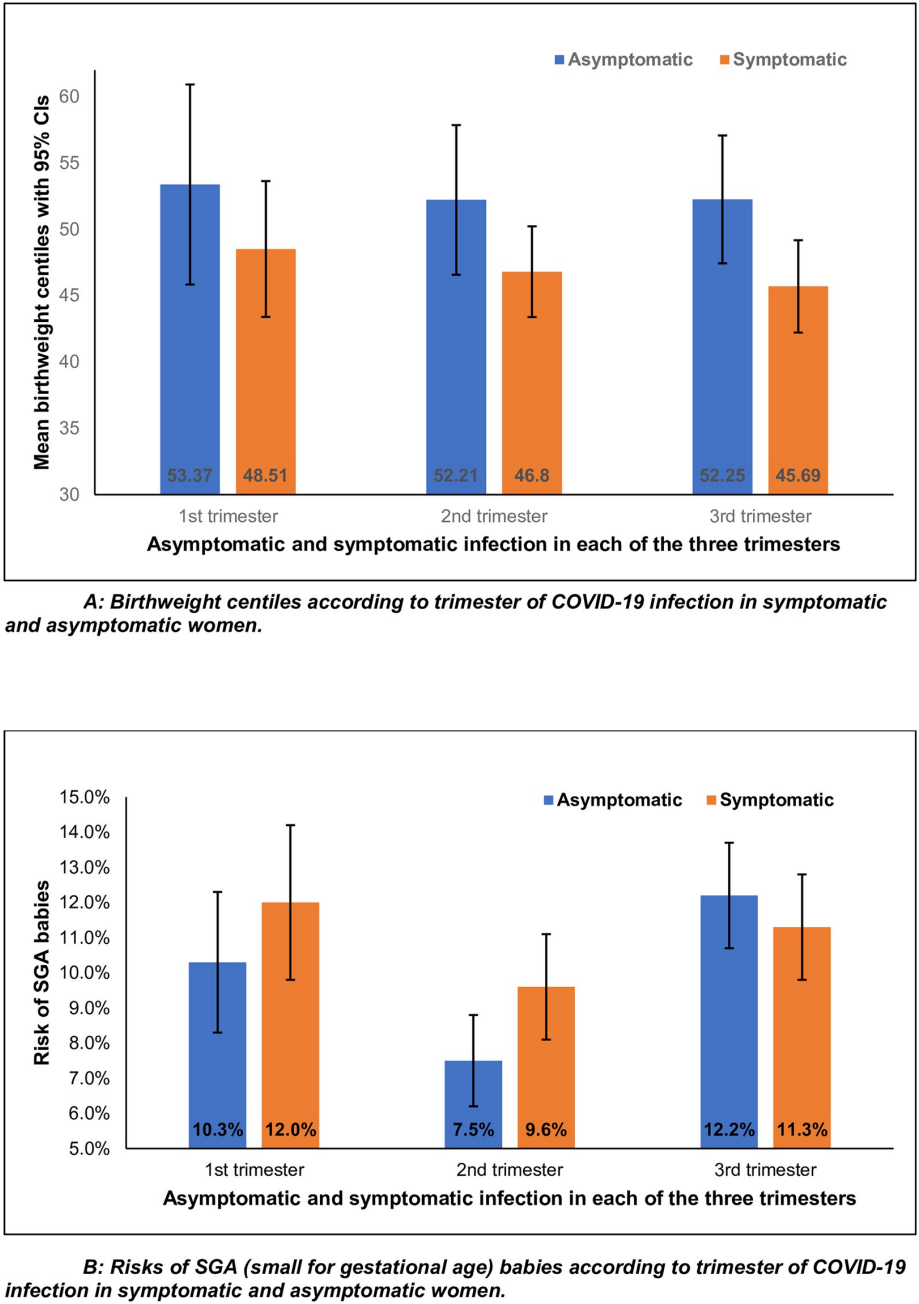
**A**. Birthweight centiles according to trimester of COVID-19 infection in symptomatic and asymptomatic women. **B**. Risks of SGA babies according to trimester of COVID-19 infection in symptomatic and asymptomatic women.

Ninety-four symptomatic women (13%) gave birth to an SGA baby compared to 10% of asymptomatic women, although the difference was not significant. Fig 1A demonstrates that in each trimester, the asymptomatic patients had higher birthweight centiles than the symptomatic women; however, there was no difference in birthweight centiles between each trimester of infection (analysed by Kruskal Wallis test, p = 0.752). When infected in the first and second trimesters, symptomatic women had a higher risk of SGA babies (12.0% vs 10.3% in first trimester, 9.6% vs 7.5% in second trimester) compared to asymptomatic women, although not statistically significant. When infected in the third trimester, the risks are similar (11.3% vs 12.2%) in the groups (Fig 1B).

In the 175 women who were infected in the first 20 weeks of pregnancy and satisfied the inclusion criteria for the secondary analysis, 132 developed symptoms (75%). The birthweight, birthweight centile and fetal growth velocity z score were compared between symptomatic and asymptomatic women within this subgroup, as shown in Table 4. Tests for normality of distribution indicated that the birthweight centile (SW 0.95, p<0.001), the Z score (SW 0.93, p<0.001) and the growth velocity (SW 0.95, p<0.001) were not normally distributed data.

**Table 4:** Comparison between symptomatic and asymptomatic in women infected with COVID-19 within 20 weeks of gestation.

Variables Total women = 175		Asymptomatic (n = 43)	Symptomatic (n = 132)	P value
**Birthweight in grams, Mean (SD)** *		3284 (468)	3061 (554)	**0.019**
**Birthweight centile, Median (Range)** **		71.2 (4.4–100)	48.2 (0–100)	**0.010**
**Estimated fetal weight in grams Mean (SD)**	**First ultrasound**	1923 (418)	1868 (517)	0.528
	**Second ultrasound**	2855 (443)	2769 (536)	0.343
**Number of days between ultrasound scans Median (Range)** **		30 (14–42)	30 (14–42)	0.768
**Daily increment in EFW in grams/day Median (Range)** **		29.5 (7.8–52.4)	29.4(-3.0–53.3)	0.413
**Standardised Z score, Median (Range)** **		0.75 (-4.1–5.2)	0.63 (-6.4–5.6)	0.368

*- analysis performed using Student t-test;

**- analysis performed using Mann Whitney U test;

SD- standard deviation; IQR- interquartile range; p<0.05 considered evidence against null hypothesis of no difference

In this subgroup, symptomatic women had significantly smaller babies (3061 gms vs 3284 gms; p = 0.019) and smaller median fetal birthweight centiles (48.2% vs 71.2%, p = 0.01) compared to the asymptomatic women. The timing of the ultrasound scans and mean estimated fetal weights in the scans were similar between the groups. The fetal growth velocity z score was lower in the symptomatic women group, although this difference did not reach statistical significance.

## Discussion

### Summary of findings

The main study results show no significant difference in birthweight, or customised birthweight centiles according to the trimester of pregnancy during which the women tested positive for COVID-19. However, symptomatic women in the whole cohort were significantly more likely to have infants with lower birthweight and mean birthweight centiles.

In the secondary analysis in the subgroup of women infected within the first 20 weeks of gestation, symptomatic women had significantly lower birthweight and birthweight centiles. They also have lower fetal growth velocity standardised z scores compared to the asymptomatic, although this did not reach statistical significance.

### Comparison with existing literature

In a large cohort study enrolling 2789 pregnant women who had COVID-19 in Israel, over the same time as this study, 17% got infected in the first trimester and 48% in the third [18]. Our study reports comparable findings of 42% of women being infected in the third trimester and the least proportion (20.5%) in the first. The rationale for looking at the timing of COVID-19 infection stems from reports showing the possibility of the placenta being affected due to maternal infection, attributed to the expression of the COVID-19 target angiotensin receptor enzyme 2 (ACE2) in the placenta [19]. Therefore, the expectation is that infection during earlier trimesters could affect the placental circulation more and lead to consequences such as preeclampsia, fetal growth restriction, abruption and preterm birth compared to infection in the third trimester. However, our study could not find evidence for this hypothesis, and COVID-19 infection in pregnancy seemed to affect the placenta equally regardless of the trimester of infection.

The SGA rate in this cohort of 12.2% is much higher than previous reports from the country, showing an SGA rate of 6% in 2017 [20]. A similar SGA rate of 15% was reported in a study of COVID-19 pregnancies from a neighbouring country in the Middle East [21]. This study reported no significant difference in the SGA risk according to the gestational age of infection, similar to our conclusion. Fallach et al. reported no significant effect of the trimester of infection on growth restriction, even though they report a higher risk of SGA than COVID-19 negative pregnancies, supporting the findings in our study [18].

The overall gestational age at delivery in our cohort is around 268 days or 38 completed weeks of gestation, a figure comparable with a recent meta-analysis [22]. The meta-analysis also reported a mean birthweight of 3144 grams in COVID-19 pregnancies, which matches our results (mean birthweight of 3171 grams). Our study further adds to the evidence that COVID-19 in pregnancy is unlikely to decrease the mean birthweight, adjusted customised birthweight centile or gestational age at birth because most pregnancies progress to term without complications. Additionally, there was no difference in the mean birthweights or adjusted centiles between the trimesters. This supports the results from a large time-series matched analysis done in England comparing the pre-COVID era with the COVID era, where similar findings are reported [23].

In this study, we were interested in the SGA risk in symptomatic COVID-19 compared to asymptomatic disease, as recent evidence shows higher odds for small for gestational age babies with symptomatic disease. A meta-analysis comparing symptomatic and asymptomatic disease (that included ten studies) showed a much higher risk of preterm birth and SGA and lower mean birthweight in symptomatic disease compared to asymptomatic [24]. Our results show that overall, the birth weight and customised birthweight centiles are lower in symptomatic disease, regardless of trimester of infection. Since gestational age at delivery and maternal demographics are included in the calculation of customised birthweight centiles and classification of SGA, increased preterm birth or any maternal demographics such as age or BMI are unlikely to be confounding factors here. In symptomatic disease, the virus proceeds to invade the cells with ACE2 and replicate rapidly, resulting in a cytokine storm- this is much less or absent in asymptomatic disease [25]. Since the placenta also expresses ACE2, it’s more likely that symptomatic disease affects the placental circulation more, pointing towards a possible association between symptomatic COVID-19 infection and SGA.

In Qatar, the risk of preterm birth is nearly 9% in the general population [26]. In this study, nearly 10.6% of the cohort had a preterm birth (slightly higher risk). This could be explained by the increased iatrogenic requirement for premature delivery in order to improve maternal recovery from severe disease. A large multinational cohort study looking at COVID-19 in pregnancy, reports a risk of 11% in asymptomatic women and 13% in symptomatic (which reached nearly 30% when the women had severe symptoms) [27]. In our study, the risks are similar regardless of symptoms as the majority had only mild symptoms.

A prospective study in Italy, published in 2021, looked at the impact of COVID on fetal growth measured as the changes in the ultrasound parameters, compared to women without COVID in pregnancy and according to the trimester of infection [28]. No significant differences were noted between the groups, and they did not support increased fetal surveillance for COVID affected pregnancies. However, they had a small sample size of 49 COVID pregnancies and did not consider disease symptoms. The results of our study point towards a possible decrease in fetal growth in symptomatic disease developing before 20 weeks of gestation (the period of pregnancy when the placental blood flow is being established and, therefore, more likely for the disease to affect fetal growth). This is shown in the significantly lesser birthweight centile in the symptomatic group. The lack of significant results in the fetal growth scores could be due to a lack of power and larger prospective studies are needed to corroborate these findings.

### Strengths and limitations

This study is the first of its kind in the country, with data collection performed by an expert team of physicians handling and interpreting electronic medical records. In addition, periodic reviews were done to ensure the adequacy and accuracy of the data collected. The birthweight outcome was recorded for all patients and was measured using similar scales in the same way by the midwives. Even though there is the possibility of human error, the birthweight is entered in multiple documents and in the official birth notification and is unlikely to be wrong. The birthweight centiles were calculated using data with no missing values, hence unlikely to have associated errors.

However, there are some limitations considering the retrospective nature of the study. The classification into symptomatic and asymptomatic is based on patient-reported symptoms- this can be subject to misclassification as patients could have possibly denied symptoms due to the social stigma and fear of isolation surrounding COVID-19. We have not done an adjusted analysis for the main exposure groups due to the lack of statistically significant results in the univariate analysis. The association between the symptom status and the perinatal outcomes were exploratory (in the main cohort and the subgroup), and further adjusted analyses in a larger cohort of women are needed. There is also a lack of data regarding potential confounders like smoking status, socioeconomic status and antenatal care that can affect the exposure and the outcome- the possibility of confounding must be kept in mind while interpreting these results.

## Conclusion

In this cohort of women infected with COVID-19 during their pregnancies, the trimester of pregnancy in which they were infected did not have an impact on perinatal outcomes such as mean birthweight, mean adjusted birthweight centiles and risk of SGA. However, these women did experience a higher risk of SGA than the nationally expected rate- implying that COVID-19 could possibly affect fetal growth through vertical transmission to the placenta regardless of the gestational age at diagnosis. Symptomatic women had lower mean birthweight and birthweight centiles and a higher risk of SGA than asymptomatic women, regardless of when they were infected. Being infected within the first 20 weeks of gestation could impact fetal growth velocity. Further larger studies are required to corroborate these findings. However, this evidence points towards changing practice and increasing fetal surveillance in women who develop COVID-19 during pregnancy to reduce neonatal morbidity.
